# CT-based multi-phase Radiomic models for differentiating clear cell renal cell carcinoma

**DOI:** 10.1186/s40644-021-00412-8

**Published:** 2021-06-23

**Authors:** Menglin Chen, Fu Yin, Yuanmeng Yu, Haijie Zhang, Ge Wen

**Affiliations:** 1 Medical Imaging teaching and research office, Nanfang hospital, Southern Medical University, No.1838 Guangzhoudadao Avenue north, Guangzhou, 510515 Guangdong China; 2 Radiology department, The second affiliated hospital of Kunming medical university, No. 374 Dianmian Road, Kunming, 650032 Yunnan China; 3 College of Electronics and Information Engineering, Shenzhen University, Shenzhen, 518068 China; 4 Department of MRI, The First People’s Hospital of Yunnan Province, The Affiliated Hospital of Kunming University of Science and Technology, No. 157 Jinbi Road, Kunming, 650032 Yunnan China; 5 Department of Radiology, Shenzhen Second People’s Hospital, No.3002, West Sungang Road, Futian District, Shenzhen, 518052 China

**Keywords:** Clear cell renal cell carcinoma, Radiomics, Improved enhanced parameters, LASSO regression

## Abstract

**Background:**

The aim of the study is to compare the diagnostic value of models that based on a set of CT texture and non-texture features for differentiating clear cell renal cell carcinomas(ccRCCs) from non-clear cell renal cell carcinomas(non-ccRCCs).

**Methods:**

A total of 197 pathologically proven renal tumors were divided into ccRCC(*n* = 143) and non-ccRCC (*n* = 54) groups. The 43 non-texture features and 296 texture features that extracted from the 3D volume tumor tissue were assessed for each tumor at both Non-contrast Phase, NCP; Corticomedullary Phase, CMP; Nephrographic Phase, NP and Excretory Phase, EP. Texture-score were calculated by the Least Absolute Shrinkage and Selection Operator (LASSO) to screen the most valuable texture features. Model 1 contains the three most distinctive non-texture features with *p* < 0.001, Model 2 contains texture scores, and Model 3 contains the above two types of features.

**Results:**

The three models shown good discrimination of the ccRCC from non-ccRCC in NCP, CMP, NP, and EP. The area under receiver operating characteristic curve (AUC)values of the Model 1, Model 2, and Model 3 in differentiating the two groups were 0.748–0.823, 0.776–0.887 and 0.864–0.900, respectively. The difference in AUC between every two of the three Models was statistically significant (*p* < 0.001).

**Conclusions:**

The predictive efficacy of ccRCC was significantly improved by combining non-texture features and texture features to construct a combined diagnostic model, which could provide a reliable basis for clinical treatment options.

## Background

Renal cell carcinoma (RCC), as the seventh most common malignant tumors in humans, categorized generally into two major groups: clear cell renal cell carcinoma (ccRCC) and non-clear cell renal cell carcinoma (non-ccRCC) [1]. Non-ccRCC mainly includes papillary RCC (pRCC) and chromophobe RCC (chRCC) [2]. ccRCC and non-ccRCC have significantly different prognoses, genetic expression patterns, and therapeutic approaches, and ccRCC has much worse prognosis and accounts for 94% of metastatic RCC [1, 2]. Furthermore, ccRCC and non-ccRCC have different responses to molecularly targeted therapies, especially in advanced and metastatic RCCs [3, 4]. Therefore, accurate classification of ccRCC and non-ccRCC before surgery or treatment has great clinical significance.

Percutaneous renal biopsy is the gold standard for the diagnosis of pathological characteristics within renal mass, but it is an invasive examination, and there may be intolerance for some elderly patients with weak constitution. Therefore, non-invasive imaging method is of great value.

Due to individual patient and technical factors, normalizing the lesion attenuation and the reference standard is essential to normalize for the iodine load and variations in relative lesion attenuation. Previous studies have shown that computed tomography (CT) and Magnetic resonance (MR) based imaging methods contribute to the differentiation of ccRCC from other subtypes [5–8], but this technique is subjective and the diagnostic accuracy still depends on the doctor’s clinical experience.

In recent years, artificial intelligence methods using Radiomic Features (RFs) analysis has gradually attracted increased attention. RFs characterize microscale information that cannot be recognized by naked human eyes, such as texture features [9, 10]. The method of RFs analysis can extract and analyze tumor information hidden within conventional medical imaging in a high-flux way to provide radiologists with more accurate image diagnostic information and decision support in the clinic regardless of the experience of clinicians [9–11]. Previous studies have demonstrated that RFs could potentially be used in cancer classification and survival prediction for different cancers such as colorectal cancer [12, 13]. Compared with those high incidence and high mortality rate tumors, texture features are less used in renal tumors, and there are still many spaces for improvement. To our knowledge, some studies did not include the entire 3D tumor tissue or four-phase images for analysis, which may lead to the loss of some tumor biological information [14, 15]. In addition, most studies have focused on the differentiation between renal malignancy and benign renal tumors, and relatively few studies have focused on the differentiation between ccRCC and non-ccRCC [16, 17]. Furthermore, few studies have incorporated the CT texture and non-texture features to construct a radiomic model.

In summary, the purpose of this study is to compare the predictive models that based on a set of CT texture and non-texture features for differentiating ccRCC from non-ccRCC.

## Methods

### Patients

This study was approved by the Ethics Committee of our hospital (approval number: NFEC-2018 − 104), and the patients’ informed consent were waived. The 197 patients in this study were obtained from the picture archiving and communication system (PACS) of our hospital from January 2013 to December 2018.

The inclusion criteria were as follows: (1) patients with no previous treatment before CT examination;(2) CT examination with no significant image noise or significant artefacts; (3) CT examination with four phases (the non-contrast phase, NCP; the corticomedullary phase, CMP; the nephrographic phase, NP and the excretory phase, EP). The exclusion criteria were as follows: (1) RCC with most or all of the cysts features;(2) patients with two or more lesions in unilateral or bilateral kidneys to avoid potential clustering effects; (3) RCC with multiple pathologic types of tumors to avoid different pathological types affect each other.

Of these cases, including 143 ccRCCs, 25 pRCCs and 29 chRCCs, 81 patients’ final pathological diagnosis were acquired from partial nephrectomy,109 from radical nephrectomy and 7 from biopsy. There were no differences in patient age or sex or maximum tumor diameter comparing ccRCCs (mean age ± standard deviation (SD), 53.202 ± 13.086 years; mean diameter ± SD, 5.283 ± 2.602 cm;92 men and 51 women;62 tumor located in the right kidney, and 81 in the left) with non-ccRCCs (52.804 ± 12.857 years; 4.929 ± 2.615 cm;31 men and 23 women; 31 tumor located in the right kidney, and 23 in the left) tumors (*p* = 0.691,0.373,0.323 and 0.923 respectively). Demographic and clinical characteristics of patients with ccRCC and non-ccRCC is summarized in Table 1.

**Table 1:** Demographic and clinical characteristics of patients with ccRCC and non-ccRCC

characteristic	ccRCC	non-ccRCC	P Value
Number	143	54	
Gender			0.323
Male	92	31	
Female	51	23	
Age (mean ± SD, years)	53.202 ± 13.086	52.804 ± 12.857	0.691
Maximum diameter (mean ± SD, cm)	5.283 ± 2.602	4.929 ± 2.615	0.373
Tumor location			0.923
Right	62	31	
Left	81	23	

### Acquisition of CT images

The data were collected from a 64-multidetector spiral CT scanner (Somatom Definition CT, siemens Medical Solutions) and a 256-multidetector spiral CT scanner (Brilliance iCT, Philips Medical Systems). The patients were in supine position, and the scanning range was from the phrenic top to the lower pole of both kidneys during breath holding. The nonionic iodine contrast agent (Iohexol, GE, American; Ultravist, Bayer, Germany) was injected by high pressure injector at a dose of 2 ml/kg and a speed of 2.5 ml/s. All the studies involved at least four-phase scanning, including CMP, PP, EP,which were started at 30-35th,60-70th,190 − 200th second after the contrast agent was injected into the antecubital vein. The scanning parameters were as follows: tube voltage 120 kV, tube current 150-320 mA, thickness of scan layer and the interlayer spacing 5 mm, field of view (FOV) 360 mm, a matrix of 512 × 512.

Image normalisation and grey level discretisation procedures were done to minimise inter-scanner effects and variations. We used the ±3 sigma technique to normalized CT image signal intensities [18, 19]. Follow this, the grey level discretisation was done by assigning a grey level range between 1 and 2 k, where k is the bits/pixel [20]. The k-value was chosen to be 6 for co-occurrence matrix features and run-length matrix features, 4 for gradient features, and 8 for wavelet features.

### Construction of non-texture features model

#### Image analysis

**Fig. 1:**
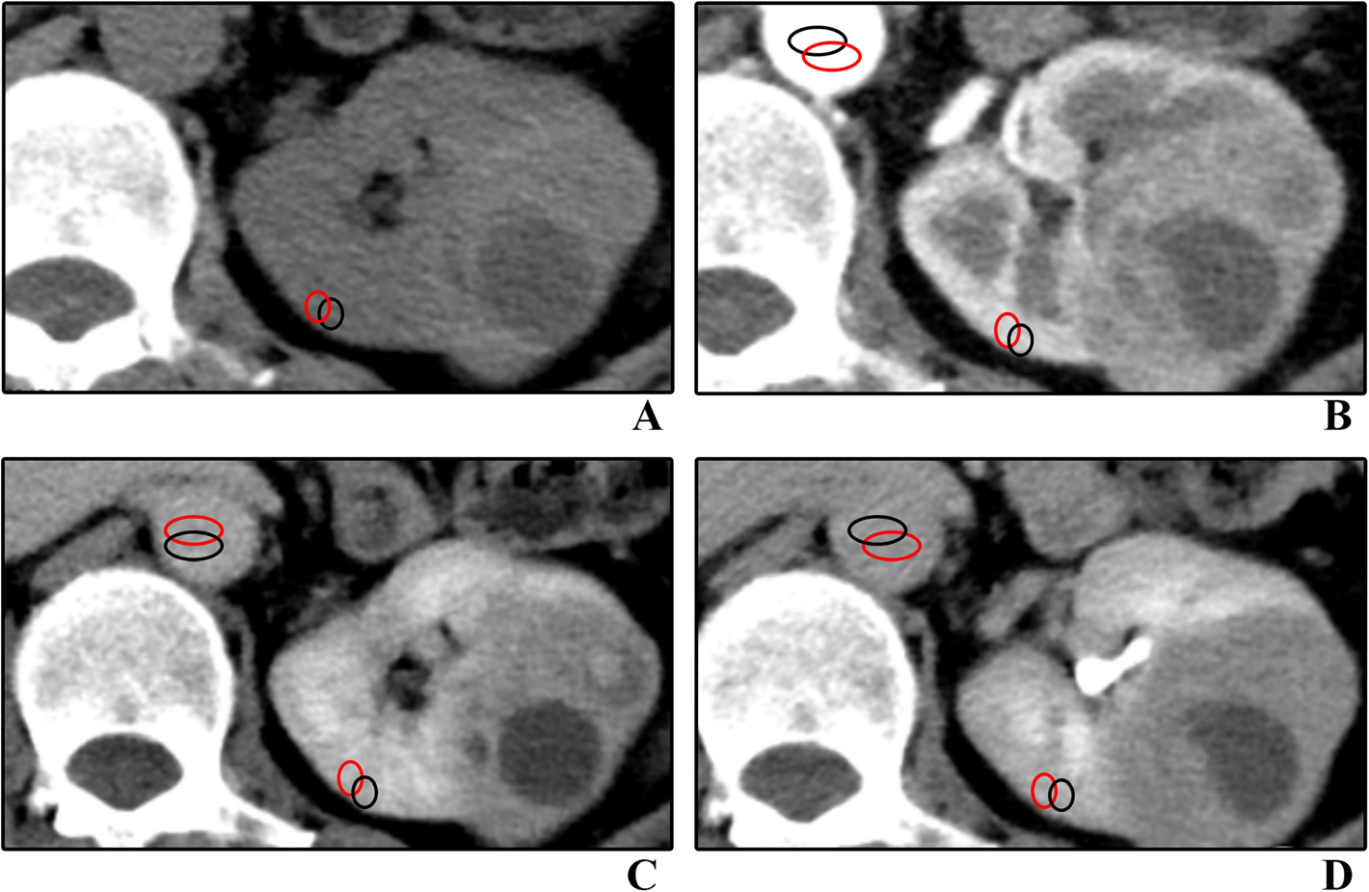
57-year-old woman with clear cell renal cell carcinoma. The red and black circles represent ROIs outlined by two radiologists including cortex ROI and aortic ROI, and the corresponding positions of ROIs in the four-phase images are the same. **A,** Unenhanced image. **B,** Corticomedullary phase image. **C,** Nephrographic phase image. **D**, Excretory phase image

This part of the data set consists of CT values of lesions. Two region of interests (ROIs) of approximately 0.5-1 cm^2^ was manually placed by two radiologists (5 and 12 years of experience) on the most avidly enhancing part of a heterogeneously enhancing lesion or in the center of a homogeneously enhancing lesion on the largest area of lesion. At the same time, necrosis, blood vessels and calcification components should be avoided. In addition, the corresponding position and size of each ROI are consistent in the four phase CT images. Finally, the lesion attenuation value in each phase was obtained by calculating the average attenuation value of the two ROIs. The attenuation value of the renal cortex and aorta were also measured as references to indicate the iodine load (Fig. 1). The enhancement values were calculated with specific formulas in Table 2.

**Table 2:** Enhancement parameters of the lesion and its formulas

Enhancement parameters	Formulas	References
Lesion Attenuation Value	–	
Absolute Deenhancement	T_3_-T_2_; T_4_-T_3_	[21]
Absolute Enhancement	T_2/3/4_-T_1_	[21]
Percentage Enhancement Ratio of Absolute Enhancement	(T_2/3/4_-T_1_)/T_1_	
Percentage Enhancement Ratio of renal cortex	C_1/2/3/4_/ T_1/2/3/4_	[22]
Percentage Enhancement Ratio of aorta	T_2/3/4_/ A_2/3/4_	[2]
Relative Attenuation of renal cortex	(T_1/2/3/4_–C_1/2/3/4_) / C_1/2/3/4_	[21]
Relative Attenuation of aorta	(A_2/3/4_–T_2/3/4_) / A_2/3/4_	
Enhancement Change of renal cortex	C_1/2/3/4_- T_1/2/3/4_	[22]
Enhancement Change of aorta	A_2/3/4_-T_2/3/4_	
Relative Enhancement Value of the Tumor (rTEV_2/3/4_)	(T_2/3/4_-T_1_)/ (C_2/3/4_-C_1_)	[2]
Corrected CT values relative to aorta	(Sa/ A_2/3/4_) *T_2/3/4_	
Corrected CT values relative to renal cortex	(Ac/ C_1/2/3/4_) *T_1/2/3/4_	

Note: T1, T2, T3 and T4 represent the CT values of the tumors in unenhanced phase, corticomedullary phase, nephrographic phase, excretory phase respectively.C1, C2, C3 and C4 represent the CT values of normal renal cortex adjacent to the tumor in unenhanced phase, corticomedullary phase, nephrographic phase, excretory phase respectively. A2, A3 and A4 represent the CT values of the aorta in corticomedullary phase, nephrographic phase, excretory phase respectively. Sa represents the mean CT values of the corresponding layers of aorta in corticomedullary phase, nephrographic phase, and excretory phase respectively. Ac represents the mean CT value of the normal renal cortex adjacent to the tumor in unenhanced phase, corticomedullary phase, nephrographic phase, and excretory phase respectively

#### Establishment of non-texture features model

The t-test or Mann-Whitney U test was performed to compare the magnitude of the above enhancement parameters of ccRCCs with non-ccRCCs in each of the four phases. A receiver operating characteristic curve (ROC) and the area under the curve (AUC) was used to evaluate the diagnostic performance of the enhanced parameters measurements. The three most discriminative parameters with *P* < 0.001 in each phase were then entered as predictors in logistic regression models, and ROC curves were computed. The t-test or Mann-Whitney U test was performed to compare differences between two groups. The AUC was used to determine the threshold with the highest accuracy in discriminating ccRCC from non-ccRCC. For each threshold level, sensitivity, specificity, positive predictive value (PPV), negative predictive value (NPV), and accuracy were calculated. Comparisons among AUCs between every two model were evaluated according to DeLong test. Matthews correlation coefficient (MCC) was also used to overcome the class imbalance issue.

### Construction of texture features model

#### Image segmentation

**Fig. 2:**
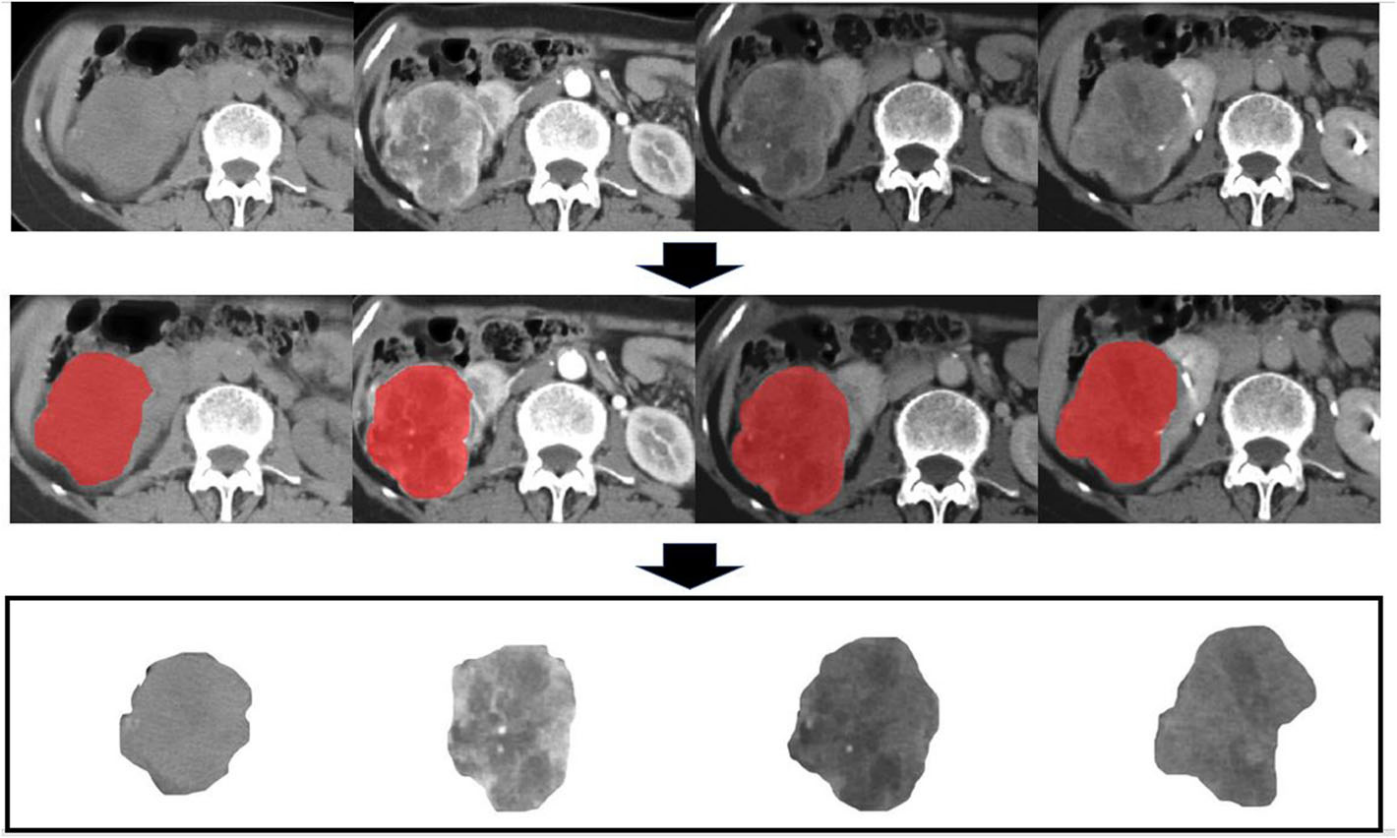
Graph shows the process of obtaining tumor tissue from original images by manual segmentation in unenhanced, corticomedullary, nephrographic, and excretory phase from left to right

Axial images of four phases (NCP, CMP, NP, and EP) with a layer thickness of 5 mm were included in this study. These original images were manually segmented by two radiologists in a laptop equipped with ITK-SNAP software (www.itk-snap.org). The entire 3D tumor tissue was segmented with margin shrinkage of 2 mm from the lesion contour to avoid the inclusion of peripheral fat and normal renal tissue [23]. The specific image segmentation process is shown in Fig. 2.

#### Texture features extraction

Texture features were extracted using PyRadiomics [11]. The segmented images were loaded and pre-processed, and over-sampling is used in order to solve the problem of data imbalance between ccRCCs and non-ccRCCs. Then texture features were extracted using the categories of gray level cooccurrence matrix, (GLCM), gray level run length matrix, (GLRLM), gray level size zone matrix, (GLSZM), neighbouring gray tone difference matrix, (NGTDM) and gray level dependence matrix, (GLDM).

#### Establishment of texture features model

We used the least absolute shrinkage and selection operator (LASSO) to select the most valuable texture features and calculated the texture score (Texture-score) for each patient via a linear combination of the selected features weighted by their corresponding coefficients. Mann-Whitney U test was performed to compare differences between texture scores of ccRCC and non-ccRCC group. The AUC was used to determine the threshold with the highest accuracy in discriminating ccRCC from non-ccRCC. For each threshold level, sensitivity, specificity, PPV, NPV, and accuracy were calculated. Comparisons among AUCs between every two model were evaluated according to DeLong test. MCC was also used to overcome the class imbalance issue.

### Construction of combined diagnostic model

On the basis of non-texture features (Model 1), and of Texture-score (Model 2), a logistic regression with those above variables was performed to build a combined diagnostic model (Model 3). For each threshold level, sensitivity, specificity, PPV, NPV, and accuracy were also calculated. Comparisons among AUCs between every two model were evaluated according to DeLong test. MCC was also used to overcome the class imbalance issue.

### Statistical analysis

All analyses were performed by using SPSS (IBM SPSS Statistics for Windows, Version 20.0. Armonk, NY: IBM Corp) and R version 3.1.0 (R Foundation). Although P<0.05 was considered to indicate a significant difference, we used a stepwise Holm-Bonferroni procedure to counteract the potential for type I errors arising from multiple comparisons. The 43 features were first ranked in ascending order of *P* value; if the first P value was less than 0.05/43, we then compared the second P value to 0.05/ (43–1), and we continued this stepwise comparison until no further *P* values indicated a significant difference.

A synthetic minority oversampling technique (SMOTE) [24] was adopted by sample generation of minority group from joint weighting of optimal features in order to tackle the adverse impact of the imbalance dataset in this study (143:54) on the performance of texture features. The synthetic cases will have attributes with values similar to the existing cases and not merely replications in order to increase the representation of the non-ccRCC group in the dataset while reflecting the structure of the original cases [25, 26].

Re-proposed by Baldi and colleagues [27] in 2000, MCC is a standard performance metric for machine learning, and was usually used to overcome the class imbalance issue through its mathematical properties invariances for class swapping. MCC now has become a successful indicator with a natural extension to the multiclass case [28]. Besides, the *F*_1_ measure is widely used in most application areas of machine learning, not only in the binary scenario, but also in multiclass cases.

$$ MCC=\frac{TP\ast TN- FP\ast FN}{\sqrt{\left( TP+ FP\right)\ast \left( TP+ FN\right)\ast \left( TN+ FP\right)\ast \left( TN+ FN\right)}} $$

$$ F1\  score=\frac{2\mathrm{TP}}{2 TP+ FP+ FN} $$

TP = Ture positives. TN = Ture negatives. FN = False negatives. FP = False positives.

## Results

### Construction of non-texture features model (Model1)

There were 0,12,13, and 8 non-texture features statistically significant for distinguishing ccRCC from non-ccRCC in NCP, CMP, NP, and EP respectively. In those non-texture features, most of the improved enhancement parameters had higher diagnostic efficacy than directly measured lesion attenuation value, and the specific results were shown in Table 3.

**Table 3:** Non-texture features of ccRCC and non-ccRCC

	ccRCC	non-ccRCC	P value	AUC
**NCP**				
Lesion Attenuation Value	33.996士9.443	35.843士7.821	0.109	0.574
Relative Attenuation of renal cortex	0.075士0.320	0.105士0.297	0.610	0.624
Percentage Enhancement Ratio of renal cortex	1.035士0.387	0.984士0.288	0.528	0.529
Enhancement Change of renal cortex	-1.862士9.447	-2.567士9.514	0.640	0.522
Corrected CT values relative to renal cortex	34.506士10.119	34.551士9.685	0.213	0.658
**CMP**				
Lesion Attenuation Value	124.394士47.847	78.494士34.898	<0.001^*^	0.779
Absolute Enhancement	90.403士49.572	42.661士31.789	<0.001^*^	0.784
Percentage Enhancement Ratio of Absolute Enhancement	3.010士2.283	1.174士0.850	<0.001^*^	0.803
Relative Attenuation of renal cortex	−0.972士0.371	−0.456士0.241	<0.001^*^	0.809
Percentage Enhancement Ratio of renal cortex	1.396士0.846	2.368士1.263	<0.001^*^	0.799
Enhancement Change of renal cortex	17.664士47.849	69.795士43.817	<0.001^*^	0.780
rTEV	0.886士0.738	0.409士0.324	<0.001^*^	0.800
Corrected CT values relative to renal cortex	128.291士52.369	81.609士36.276	<0.001^*^	0.787
Corrected CT values relative to aorta	127.631士45.998	81.623士37.425	<0.001^*^	0.787
Percentage Enhancement Ratio of aorta	0.439士0.153	0.287士0.138	<0.001^*^	0.777
Relative Attenuation of aorta	0.571士0.157	0.722士0.135	<0.001^*^	0.777
Enhancement Change of aorta	174.474士73.398	215.335士85.426	0.004^*^	0.733
**NP**				
Lesion Attenuation Value	91.727士22.870	73.092士23.618	<0.001^*^	0.725
Absolute Enhancement	57.735士23.909	37.259士21.359	<0.001^*^	0.755
Absolute Deenhancement	−32.674士34.765	−5.400士20.700	<0.001^*^	0.742
Percentage Enhancement Ratio of Absolute Enhancement	1.913士1.213	1.074士0.626	<0.001^*^	0.756
Relative Attenuation of renal cortex	−0.322士0.187	−0.431士0.181	<0.001^*^	0.712
Percentage Enhancement Ratio of renal cortex	1.627士0.623	2.366士1.263	<0.001^*^	0.699
Enhancement Change of renal cortex	45.249士22.876	59.712士28.872	0.001^*^	0.660
rTEV	0.575士0.281	0.393士0.227	<0.001^*^	0.729
Corrected CT values relative to renal cortex	93.596士24.267	74.648士24.734	<0.001^*^	0.735
Corrected CT values relative to aorta	91.923士18.694	73.195士21.801	<0.001^*^	0.751
Percentage Enhancement Ratio of aorta	0.753士0.150	0.593士0.188	<0.001^*^	0.761
Relative Attenuation of aorta	0.258士0.154	0.419士0.184	<0.001^*^	0.761
Enhancement Change of aorta	30.156士19.672	50.440士23.580	<0.001^*^	0.750
**EP**				
Lesion Attenuation Value	72.978士16.014	63.385士16.952	<0.001^*^	0.670
Absolute Enhancement	38.995士16.019	27.550士14.126	<0.001^*^	0.730
Absolute Deenhancement	−18.759士12.282	−9.701士11.864	<0.001^*^	0.704
Percentage Enhancement Ratio of Absolute Enhancement	1.294士0.806	0.793士0.418	0.077	0.602
Relative Attenuation of renal cortex	−0.388士0.134	−0.468士0.134	0.341	0.555
Percentage Enhancement Ratio of renal cortex	1.724士0.550	2.034士0.600	0.678	0.524
Enhancement Change of renal cortex	45.673士16.017	56.313士21.001	0.789	0.515
rTEV	0.467士0.191	0.339士0.156	0.143	0.584
Corrected CT values relative to renal cortex	74.002士15.976	64.228士15.503	0.011^*^	0.647
Corrected CT values relative to aorta	73.050士12.505	63.274士13.974	0.016^*^	0.639
Percentage Enhancement Ratio of aorta	0.784士0.133	0.676士0.152	<0.001^*^	0.729
Relative Attenuation of aorta	0.222士0.137	0.331士0.158	<0.001^*^	0.729
Enhancement Change of aorta	20.779士13.349	31.112士14.534	<0.001^*^	0.718

Note: * represents statistically significant; the non-contrast phase, NCP; the corticomedullary phase, CMP; the nephrographic phase, NP and the excretory phase, EP

Three most discriminative parameters in each phase were then entered as predictors in logistic regression models to construct three diagnostic models in CMP, NP and EP phase respectively. Since all the variables of the NCP had no significant statistical significance for the identification of ccRCC and non-ccRCC, the diagnostic model of the NCP was not established. Finally, three different non-texture features models were constructed.

The Model 1(CMP) incorporated the three most common features identified in CMP (*Percentage Enhancement Ratio of Absolute Enhancement, Relative Attenuation of renal cortex,* and *rTEV*). The Model 1(NP) incorporated the three most common features identified in NP (*Percentage Enhancement Ratio of Absolute Enhancement, Relative Attenuation of aorta* and *Percentage Enhancement Ratio of aorta)*. The Model 1(EP) incorporated the three most common features identified in EP (*Percentage Enhancement Ratio of aorta, Absolute Enhancement,* and *Relative Attenuation of aorta)*. There were statistically significant differences between the diagnostic models of CMP, NP and EP in distinguishing ccRCC and non-ccRCC (*P* < 0.001).

### Performance of model 1 for distinguishing ccRCC

**Fig. 3:**
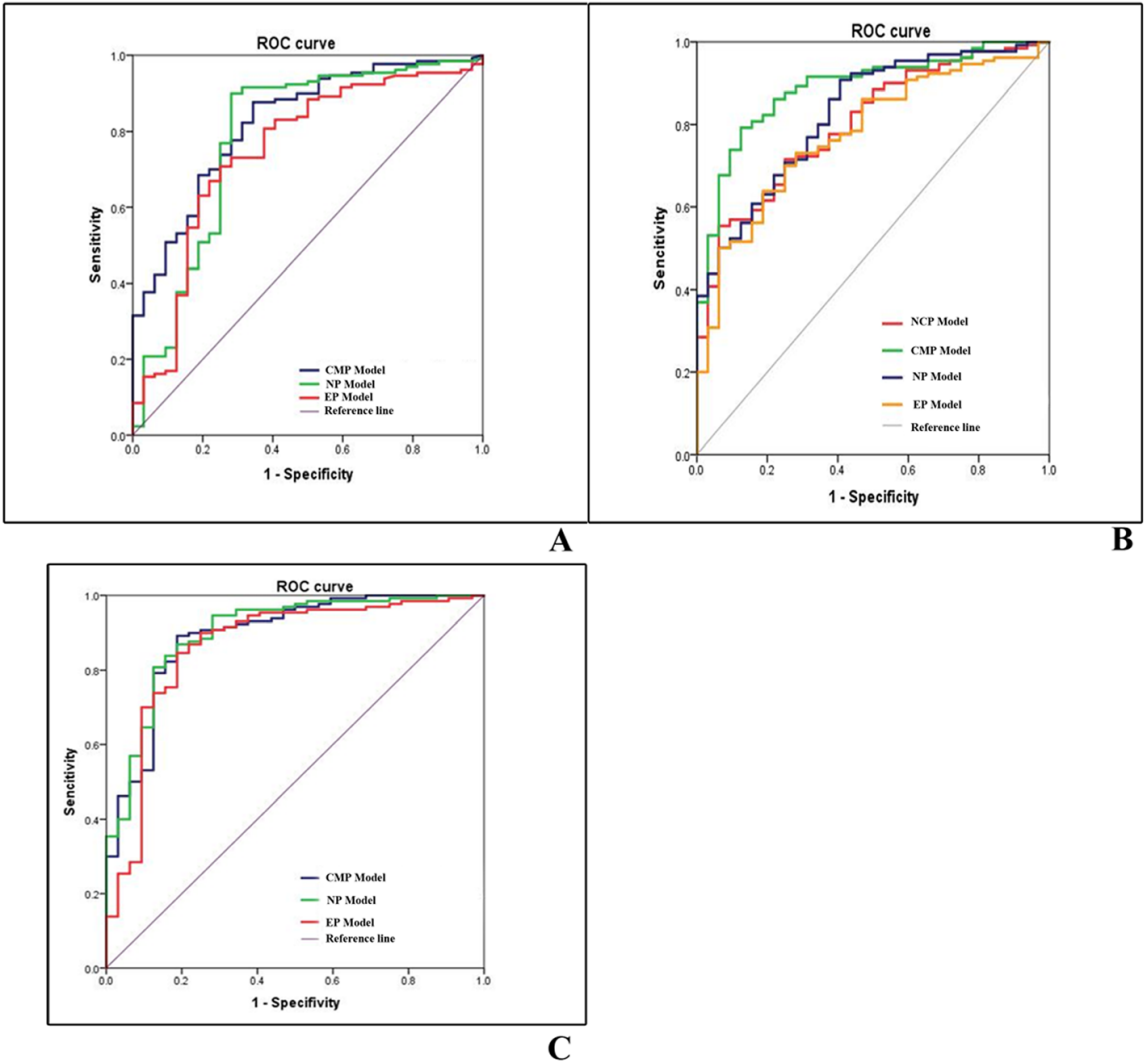
Graph shows ROC curves for differentiation of ccRCCs and non-ccRCCs on basis of non-texture features(A), texture features(B), and combined featuers(C)

The AUC and MCC value of the CMP was the highest among the three models, reaching 0.823(95% Confidence interval(CI):0.745–0.901), and + 0.557. However, the accuracy and F_1_ score of the NP were the highest, which were 0.812 and 0.844 respectively. The ROC curves results were shown in Fig. 3A.

DeLong test results showed that there was no statistically significant difference between the model in NP and model in EP(*P* = 0.120). In other words, the AUC value of model in CMP was higher than that of NP and EP (*P* = 0.032; *P* = 0.006).

### Construction of texture features model (Model2)

There were 74 features been extracted from every single phase of CT scanning, therefore, the total number of the features extracted was 296 per lesion. Those features were: 23 GLCM features, 16 GLSZM features, 16 GLRLM features, 14 GLDM features, and 5 NGTDM features. The texture features and formulas of the diagnostic Model 2 in each phase were shown in Table 4.

**Table 4:** Features and formulas for the identification of ccRCC and non-ccRCC

Features	Formulas
f47: GLCM Maximum Probability	max(*P*(i, j))
f49: GLCM Joint Energy	${\sum}_{i=1}^{N_g}{\sum}_{j=1}^{N_g}{\left(P\left(i,j\right)\right)}^2$
f56: GLCM Correlation	$\frac{\sum_{i=1}^{N_g}{\sum}_{j=1}^{N_g}\mathrm{P}\left(\mathrm{i},\mathrm{j}\right)\mathrm{ij}-{\mu}_x{\mu}_y}{\sigma_x(i){\sigma}_y(j)}$
f58: GLCM Sum Entropy	${\sum}_{k=2}^{2{N}_g}{\mathrm{P}}_{x+y}(k){\log}_2\left({\mathrm{P}}_{x+y}(k)+\varepsilon \right)$
f94: GLRLM Long Run High Gray Level Emphasis	$\frac{\sum_{i=1}^{N_g}{\sum}_{j=1}^{N_g}\mathrm{P}\left(\mathrm{i},\mathrm{j}\right){i}^2{j}^2}{\sum_{i=1}^{N_g}{\sum}_{j=1}^{N_g}\mathrm{P}\left(\mathrm{i},\mathrm{j}\right)}$
f102: GLSZM Gray Level Non Uniformity Normalized	$\frac{\sum_{i=1}^{N_g}{\left({\sum}_{j=1}^{N_g}P\left(i,j\right)\right)}^2}{N_z^2}$

The 296 features were included in the LASSO regression model to screen the most valuable features. It is known that tuning parameter(λ)corresponds to various diagnostic characteristic curves. The optimal λ selection criteria contain the minimum standard and 1-SE. We found that through preliminary research results the difference in AUC values of the two models selected by the above two criteria were very small. To construct a more concise model, λ was selected by 1-SE standard.

Finally, Texture-scores were obtained by LASSO regression. The significance of each texture parameter and texture-score in distinguishing ccRCC from non-ccRCC is summarized in Table 5.

**Table 5:** Texture-score and texture features at the NCP, CMP, NP and EP to distinguish ccRCC from non-ccRCC

Phase	Texture-score and features	ccRCC	non-ccRCC	P value
NCP	f47	0.513士0.136	0.625士0.141	<0.001
	f56	0.287士0.094	0.196士0.119	<0.001
	f58	1.582士0.265	1.344士0.299	<0.001
	f94	53.046士37.244	76.842士56.910	0.026
	Texture -score	1.704士0.538	1.115士0.551	<0.001
CMP	f49	0.095士0.057	0.193士0.080	<0.001
	Texture -score	1.723士0.471	0.810士0.677	<0.001
NP	f49	0.147士0.064	0.241士0.095	<0.001
	f102	0.224士0.056	0.302士0.086	<0.001
	Texture -score	1.571士0.287	1.123士0.415	<0.001
EP	f56	0.440士0.146	0.319士0.133	<0.001
	f58	2.335士0.360	1.994士0.274	<0.001
	Texture -score	1.568士0.364	1.212士0.270	<0.001

### Performance of model 2 for distinguishing ccRCC

Among the four models, the AUC,accuracy,MCC of the Texture -score(CMP) were the highest, which were 0.887, 0.809, and 0.601 respectively; the F_1_ score of the Model 2(NP)reached 0.838. By comparison of AUC values among models at four phases, the results showed that the difference between four phases was not statistically significant (*P* = 0.07–0.838). Specific results are shown in Table. 6 and Fig. 3B.

**Table 6:** Diagnostic performance of Model1, Model 2 and Model 3 for distinguishing ccRCC from non-ccRCC

Phase	Model	Cut-off value	AUC(95%CI)	Sensitivity	Specificity	PPV	NPV	Accuracy	MCC	F_**1**_ score
**CMP**	Model1	0.639	0.823 (0.745–0.901)	0.797	0.759	0.903	0.600	0.787	+ 0.557	0.791
	Model 2	1.500	0.887 (0.828–0.945)	0.875	0.792	0.515	0.963	0.809	+ 0.601	0.835
	Model 3	0.718	0.891 (0.824–0.957)	0.892	0.812	0.953	0.656	0.877	+ 0.637	0.936
**NP**	Model1	0.302	0.784 (0.680–0.889)	0.881	0.630	0.867	0.675	0.812	+ 0.548	0.844
	Model 2	1.179	0.826 (0.752–0.901)	0.908	0.594	0.869	0.507	0.803	+ 0.598	0.838
	Model 3	0.853	0.900 (0.837–0.963)	0.808	0.875	0.961	0.538	0.821	+ 0.625	0.897
**EP**	Model1	0.927	0.748 (0.647–0.849)	0.664	0.759	0.883	0.462	0.690	+ 0.297	0.718
	Model 2	1.362	0.776 (0.694–0.859)	0.700	0.750	0.915	0.384	0.710	+ 0.376	0.759
	Model 3	0.737	0.864 (0.783–0.945)	0.846	0.812	0.953	0.571	0.840	+ 0.627	0.915

### Construction of combined diagnostic model (Model3)

The results of the two-class multivariate logistic regression model showed that there were 2 (Texture-score, Enhancement Change of aorta), 4 (Texture-score, Absolute Deenhancement, Corrected CT values relative to aorta, and Enhancement Change of aortia), and 2 (Texture-score, Enhancement Change of aortia) independent factors in distinguishing ccRCC from non-ccRCC in CMP, NP, and EP, respectively. In addition, our results showed that the Texture-score in three enhanced phases were independent risk factors for ccRCC diagnosis, that is, the higher the value of the Texture-score, the higher the possibility of clinical indication of ccRCC. Among them, the top two features with the strongest correlation were the Texture-score in NP and EP. Non-texture features were protective factors of ccRCC (odds ratio value<1), and the correlation was far lower than texture features. Specific results were shown in Table 7.

**Table 7:** Multivariate analysis results of combined models in the differentiation of ccRCC from non-ccRCC

	Independent influencing factor	Regression coefficients	OR value	P value
CMP	Texture-score	2.654	14.205	<0.001
	Enhancement Change of aorta	−0.012	0.988	0.001
NP	Texture-score	4.114	61.187	<0.001
	Absolute Deenhancement	−0.003	0.971	0.01
	Corrected CT values relative to aorta	−0.104	0.901	0.012
	Enhancement Change of aorta	−0.120	0.887	0.002
EP	Texture-score	3.569	35.489	<0.001
	Enhancement Change of aorta	−0.075	0.928	<0.002

### Performance of model 3 for distinguishing ccRCC

In combined diagnosis model of three phases, the AUC value of the NP was the highest, reaching 0.900(95%CI:0.837–0.963), followed by the CMP and EP, while the MCC and F_1_ score of the CMP were the highest, which were 0.637 and 0.936, respectively. The DeLong test showed no significant difference in AUC values between models in CMP, NP and EP (*P* = 0.085–1). The specific results are shown in Table 6 and Fig. 3C.

### Comparison of diagnostic efficacy between model 1, model 2 and model 3

The study showed that the AUC value of the Model 3 was the highest in any of the three enhanced phases, followed by the Model 2, and Model 1. DeLong test results showed that there were significant differences in AUC values between Model 1 and 2, Model 2 and 3, Model 1 and 3 in any of the three enhanced phases (*P* < 0.001).

**Fig. 4:**
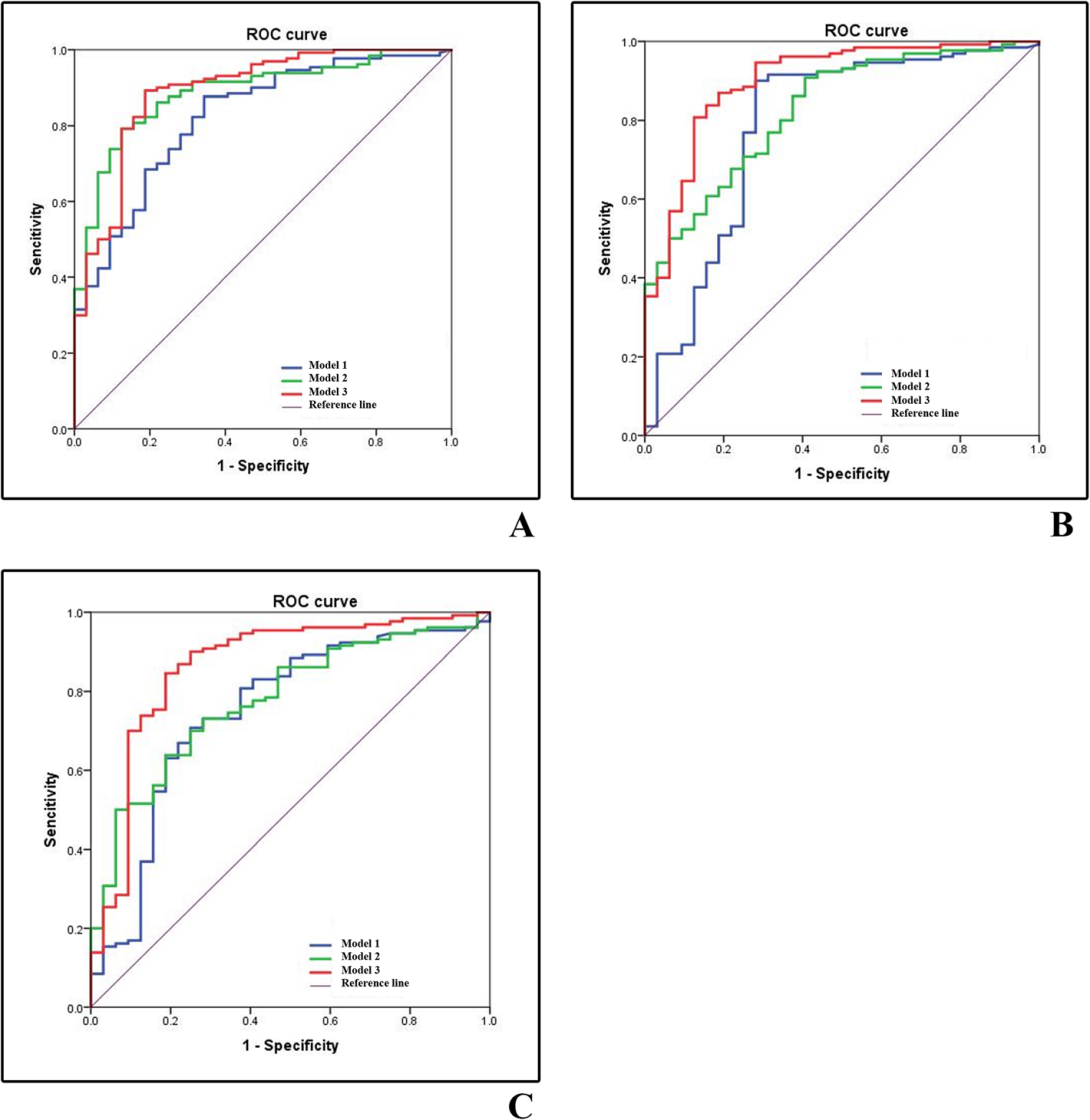
Graphs show ROC curves for differentiation of ccRCCs and non-ccRCCs of Model 1, Model 2, and Model 3. **A,** Corticomedullary phase, CMP. **B,** Nephrographic phase, NP. **C**, Excretory phase, EP

The diagnostic performance of CMP in Model 1 was the highest when compared to that in NP and EP, and the differences was statistically significant. In both Model 2 and Model 3, there were no statistically significant differences in the detection efficiency of ccRCC between CMP, NP and EP, which suggested that the diagnostic performance in NP and EP were considered to be consistent with that in CMP. The specific results are shown in Table 6 and Fig. 4.

## Discussion

In this study, we compared the diagnostic performance of models that based on a set of CT texture and non-texture features to differentiate ccRCC from non-ccRCC. In summary, we found that the model with both texture features and non-texture features could stratify patients with higher diagnostic performance. Specifically, textural features related to Maximum Probability, Joint Energy, Correlation, Sum Entropy, Long Run High Gray Level Emphasis and Gray Level Non-uniformity Normalized are the strongest predictors of ccRCC.

Previous studies had indicated the value of radiological imaging in differentiating the ccRCC from other subtypes [22, 29, 30]. Despite some positive results, there were similarities in attenuation between the RCC subtype, especially for small renal lesion [31]. Besides, due to the individual differences in heart and kidney function among different patients, there is a certain degree of deviation between the degree of renal tumor enhancement measured artificially and the actual degree of tumor enhancement. By applying mathematical methods to reduce this bias, the enhancement values, such as Absolute Enhancement, and rTEV, would be helpful for improving confidence in diagnosis and facilitating accurate diagnosis. Like the study conducted by Young et al. [2] and others, the results showed high utility of enhancement values at multiphasic multidetector CT with regard to differentiation of ccRCC from pRCCs, chRCCs, and its benign mimics [2, 21, 31–37]. In our study, 43 enhancement values were concluded as non-texture features. To the best of our knowledge, Enhancement Change of aorta and Corrected CT values relative to renal cortex has not been reported yet. We found that the diagnostic performance of most enhancement values at CMP, NP and EP in differentiating ccRCC from non-ccRCC was higher than lesion attenuation value did.

Although multiphasic CT has demonstrated useful in differentiation of RCC subtypes [8, 38], various studies have been done on assessing the radiomics features for differentiating RCC subtypes. Feng et al. [16] collected 52 cases of fat-poor angiomyolipoma and ccRCC less than 4 cm, and extracted CT texture features of the largest layer of tumor in NCP, CMP and NP. The results showed that the texture analysis method based on machine learning was conducive to the identification of the two, with an accuracy of 93.9% and AUC of 0.955.

On this foundation, we have tried to move deeper. We extracted texture features and non-texture features to construct combined diagnosis models in order to improve the diagnostic performance, so as Jiule Ding did. Jiule Ding et al. [39] retrospectively extracted CT texture features of 114 ccRCCs in the CMP and NP, and established the non-texture features diagnosis model, texture features diagnosis model and combined diagnosis model. The results showed that the combined diagnosis model had the highest diagnostic efficiency in discrimination of the high from low grade ccRCC with the AUC values of 0.878 in the training cohort. The difference is that we did this research in another aspect. To the best of our knowledge, Texture-features based models with the non-texture features is rarely used in the identification of ccRCC and non-ccRCC. In the area of distinguishing ccRCC from non-ccRCC, Zhi-Cheng Li et al. [40] extracted the RFs of the 3D tumor in the CMP and NP, and concluded that the model in CMP achieved an AUC of 0.949 and an accuracy of 92.9%. Although this article got better results, we covered more texture features in all NCP, CMP, NP and EP, and the results were more comprehensive and reliable. We also compared the diagnostic efficacy of three different diagnostic models in each phase, paving the way for further research and clinical application, which Zhi-Cheng Li did not do. Most importantly, Zhi-Cheng Li does not combine texture features with non-texture features, which is rarely used in previous study of identification ccRCC from non-ccRCC. We not only combined, but also mathematically improved the non-ccRCC feature algorithm to eliminate the individual differences.

Many researchers think the most reasonable performance metric is the accuracy, which refers to ratio between the number of correctly classified samples and the overall number of samples [41]. However, when the dataset is unbalanced, which is often seen in previous study, accuracy cannot be considered a reliable measure anymore, because it fail to consider the ratio between positive and negative elements [42–45]. Based on these reasons, this paper put forward MCC in addition to accuracy. To our knowledge, these parameters have been rarely mentioned in previous studies.

In this study, combined models of different phases were constructed, which indicates the value of texture features in the predictive model. Although Ding J et al. [39] declared that non-texture features had limited predictive value in discriminating the high nuclear grade from the low one. The Texture-score combined with non-texture features in our study improved the capacity of the prediction models for identify ccRCC. However, in other directions, the role of non-textural features needs to be further studied.

This study has the following innovations: First, we include entire 3D tumor tissue and four-phase images to analyze tumor texture features as fully as possible. Second, we applied the improved enhancement parameter based on the traditional CT value measurement method. This kind of non-texture features are rarely reported in previous studies, which is partly proposed for the first time, and we found that those non-texture features could identify ccRCC with higher diagnostic performance. Moreover, models combined Texture-score with non-texture features were also proposed in this research, which is rarely reported in literature. We also found that the combined diagnostic model was more effective than any single diagnostic model. Finally, F_1_ and accuracy generate reliable results only when applied to balanced datasets, and produce misleading results when applied to imbalanced cases. MCC, instead, was used in our research to overcome the class imbalance issue. However, there were several limitations to our study. First, this study was based on single center. In hence, large, multicenter and prospective studies should be involved in future to validate the model. Another limitation of this study is the relatively unbalanced data set. Two-thirds of the data in this study consisted of ccRCC, while this was similar in size to previous studies regarding these relatively common renal lesions [46, 47]. In the process of data preprocessing, SMOTE was used to solve the problem of data imbalance as much as possible, but further analysis is still necessary to confirm our results. Besides, previous studies have shown the value of multiparametric MRI in ccRCC differentiation [7, 48]. Incorporating features derived from these MR modalities may potentially improve the model performance.

## Conclusion

In conclusion, our results show that quantitative CT texture features along with non-texture features can be used to accurately differentiate ccRCC from non-ccRCC on CMP, NP, and EP. Our preliminary results reveal that the combination of texture feature model, and non-texture feature model constitute an optimal pipeline method preoperatively. However, this technique requires further validation on a larger scale prior to implementation into clinical practice.

## Data Availability

The datasets during and/or analyzed during the current study available from the corresponding author on reasonable request pending the approval of the institution and trial/study investigators who contributed to the dataset.

## Abbreviations

ccRCC: clear cell renal cell carcinoma
non-ccRCC: non-clear cell renal cell carcinoma
NCP: Non-contrast Phase
CMP: Corticomedullary Phase
NP: Nephrographic Phase
EP: Excretory Phase
LASSO: Least Absolute Shrinkage and Selection Operator
AUC: Area under receiver operating characteristic curve
RCC: Renal cell carcinoma
pRCC: papillary renal cell carcinoma
chRCC: chromophobe renal cell carcinoma
CT: Computed Tomography
MR: Magnetic Resonance
RFs: Radiomic features
PACS: Picture archiving and communication system
SD: Standard deviation
FOV: Field of view
ROI: Region of interest
PPV: Positive predictive value
NPV: Negative predictive value
GLCM: Gray level cooccurrence matrix
GLRLM: Gray level run length matrix
GLSZM: Gray level size zone matrix
NGTDM: Neighbouring gray tone difference matrix
GLDM: Gray level dependence matrix
CI: Confidence interval
MCC: Matthews correlation coefficient
